# Mouth-clicks used by blind expert human echolocators – signal description and model based signal synthesis

**DOI:** 10.1371/journal.pcbi.1005670

**Published:** 2017-08-31

**Authors:** Lore Thaler, Galen M. Reich, Xinyu Zhang, Dinghe Wang, Graeme E. Smith, Zeng Tao, Raja Syamsul Azmir Bin. Raja Abdullah, Mikhail Cherniakov, Christopher J. Baker, Daniel Kish, Michail Antoniou

**Affiliations:** 1 Department of Psychology, Durham University, Science Site, Durham, United Kingdom; 2 Department of Electronic Electrical and Systems Engineering, School of Engineering, University of Birmingham, Edgbaston, Birmingham, United Kingdom; 3 School of Information and Electronics, Beijing Institute of Technology, Beijing, China; 4 College of Electronic Science and Engineering, National University of Defense Technology, Changsha, China; 5 Department of Electrical & Computer Engineering, The Ohio State University, Columbus, Ohio, United States of America; 6 Department of Computer and Communication Systems Engineering, Universiti Putra Malaysia (UPM), Serdang, Selangor, Malaysia; 7 World Access for the Blind, Placentia, California, United States; University of California at Berkeley, UNITED STATES

## Abstract

Echolocation is the ability to use sound-echoes to infer spatial information about the environment. Some blind people have developed extraordinary proficiency in echolocation using mouth-clicks. The first step of human biosonar is the transmission (mouth click) and subsequent reception of the resultant sound through the ear. Existing head-related transfer function (HRTF) data bases provide descriptions of reception of the resultant sound. For the current report, we collected a large database of click emissions with three blind people expertly trained in echolocation, which allowed us to perform unprecedented analyses. Specifically, the current report provides the first ever description of the spatial distribution (i.e. beam pattern) of human expert echolocation transmissions, as well as spectro-temporal descriptions at a level of detail not available before. Our data show that transmission levels are fairly constant within a 60° cone emanating from the mouth, but levels drop gradually at further angles, more than for speech. In terms of spectro-temporal features, our data show that emissions are consistently very brief (~3ms duration) with peak frequencies 2-4kHz, but with energy also at 10kHz. This differs from previous reports of durations 3-15ms and peak frequencies 2-8kHz, which were based on less detailed measurements. Based on our measurements we propose to model transmissions as sum of monotones modulated by a decaying exponential, with angular attenuation by a modified cardioid. We provide model parameters for each echolocator. These results are a step towards developing computational models of human biosonar. For example, in bats, spatial and spectro-temporal features of emissions have been used to derive and test model based hypotheses about behaviour. The data we present here suggest similar research opportunities within the context of human echolocation. Relatedly, the data are a basis to develop synthetic models of human echolocation that could be virtual (i.e. simulated) or real (i.e. loudspeaker, microphones), and which will help understanding the link between physical principles and human behaviour.

## Author summary

Echolocation is the ability to use sound-echoes to infer spatial information about the environment. It is well known from certain species of bats or marine mammals. Remarkably, some blind people have developed extraordinary proficiency in echolocation using mouth-clicks. Human echolocation work has built on scant theoretical foundations to date. The current report characterizes the transmission (i.e. mouth click) that people use for echolocation, and in this way provides data that can be used to advance the field in a theory guided way. We collected a large database of mouth clicks with three blind people expertly trained in echolocation. This allowed us to perform unprecedented analyses. Specifically, the current report provides the first ever description of the beam pattern of human expert echolocation transmissions, as well as spectro-temporal descriptions at a level of detail not available before. Based on our measurements we also propose a mathematical model to synthesize transmissions. Thus, the data are a basis to develop synthetic models of human echolocation, which are essential for understanding characteristics of click echoes and human echolocation behaviour in tasks such as localising or recognising an object, navigating around it etc.

## Introduction

Echolocation is the ability to use sound reverberation to get information about the distal spatial environment. It has long been established that certain species of bats or marine mammals use echolocation, e.g. to navigate and locate prey [1]. Research has also demonstrated that humans are capable of echolocation [2–4]. In fact, there are some blind people who have trained themselves to use mouth-clicks to achieve extraordinary levels of echolocation performance, in some cases rivalling performance of bats [5]. Human echolocation is a biosonar system, and thus relies on both signal transmission (mouth-click) and signal reception (the ears). Head related transfer functions (e.g. HRTF data bases) can be used to model characteristics of signal reception. But, to date there is no description of transmitted mouth clicks other than approximations of their duration or peak frequencies in the straight ahead direction [6,7,8]. For the current report, we collected a large database of click emissions with three blind people expertly trained in echolocation, which allowed us to perform unprecedented analyses. Specifically, here we provide the first ever descriptions of acoustic properties of human expert echolocation clicks in the spatial domain (i.e. the emission beam pattern), as well as descriptions in spectral and time domains at a level of detail not previously available in the literature [6,7,8]. We also provide model fits to our measurements, and introduce a method to synthesize artificial clicks at various positions in space and for each of our three expert echolocators. Combined with existing HRTF databases this can be used for synthetic echo-acoustics. The data we present here open avenues for future research. For example, in bats, the spatial distribution of emissions have been used to formulate and test model based hypothesis about behaviour [9,10] and similar might be possible in humans. Also, the question arises if people may adapt their emissions pending situational demands, as it has been observed in bats [9–16]. Relatedly, the data are a basis to develop synthetic models of human echolocation that could be virtual (i.e. simulated) or real (i.e. loudspeaker, microphones), and which will help understanding the link between physical principles and human behaviour. For example, understanding characteristics of click echoes from various objects could be used to understand human echolocation behaviour in tasks such as localising or recognising an object, navigating around it etc. To undertake this type of work large amounts of data are required (for example, a radar reflectivity measurement of a single object typically requires thousands of measurements), which are impractical to ask from human subjects, and where synthetic models are needed. In the following sections we describe our measurement set-up, data analysis and results. We finish with the description of click synthesis, before discussion of limitations and implications of our work.

## Methods

The experiment was conducted following the British Psychological Society (BPS) code of practice and according to the World Medical Organization Declaration of Helsinki. All procedures had been approved by the Durham University department of Psychology ethics committee. Participants volunteered to take part in the study. Information and consent forms were provided in an accessible format, and we obtained informed consent from all participants.

### Participants

Three blind people with expertise in echolocation participated. EE1: male, 49 years at time of testing; enucleated in infancy because of retinoblastoma; reported to have used echolocation on a daily basis as long as he can remember. EE2: male, 33 years at time of testing; lost sight aged 14 years due to optic nerve atrophy; reported to have used echolocation on a daily basis since he was 15 years old. EE3: male, 31 years at time of testing; lost sight gradually from birth due to Glaucoma; since early childhood (approx 3 yrs) only bright light detection; reported to have used echolocation on a daily basis since he was 12 years old. All participants had normal hearing as assessed via pure tone audiometry (250-6000Hz). EE1 through EE3 use echolocation to go about their daily life, including activities such as hiking and travelling unfamiliar cities, playing ball and riding bicycles. There are also previous data on echo-acoustic angular resolution for EE1-EE3. EE1 and EE2 had previously taken part in a 2-interval 2-alternative forced choice echo-acoustic localization test [17] and had obtained 75% thresholds of 4° and 9°, respectively (for method details see [17]). All participants had also taken part in an echo-acoustic Vernier acuity test [5] and had obtained thresholds of 1.4°, 7.6° and 1.2°, respectively (for details see [5]).

### Set-up and apparatus

The experiment was conducted in a sound-insulated and echo-acoustic dampened room (approx. 2.9m x 4.2m x 4.9m, 24dBA noise-floor; lined with acoustic foam wedges that effectively absorb frequencies above 315 Hz). Participants were positioned in the centre of the room. The elevation of a participant’s mouth with respect to the floor was: EE1: 154cm. EE2: 170cm. EE3: 143cm. The floor was covered with foam baffles.

Recordings were made with DPA SMK-SC4060 miniature microphones (DPA microphones, Denmark) (with protective grid removed) and TASCAM DR100-MKII recorder (TEAC Corporation, Japan) at 24bit and 96kHz. A reference microphone was placed 50cm in front of the participant, at mouth level, whilst the other microphone was moved around the participant to capture variation in clicks as a function of azimuth and elevation. In the horizontal plane (mouth level) we measured a span of 270° in 10° steps starting to the right of the participant at both 40cm and 100cm distance. In the vertical plane we measured a span of 260° in 10° steps starting 40° below the mouth level plane to the front at 40cm distance.

Participants were not allowed to move their head during recording so as not to introduce error into microphone placements, as these were done with respect to the mouth. To achieve this we used a custom made set of tactile markers so that participants could move in between trials, but could reliably place their head in the correct position and orientation for recording.

### Instruction to participants

Participants were instructed to make clicks as they normally would in their daily life. The room was empty except for the microphones, and the participants knew this.

### Description/Analysis of clicks

**Fig 1:**
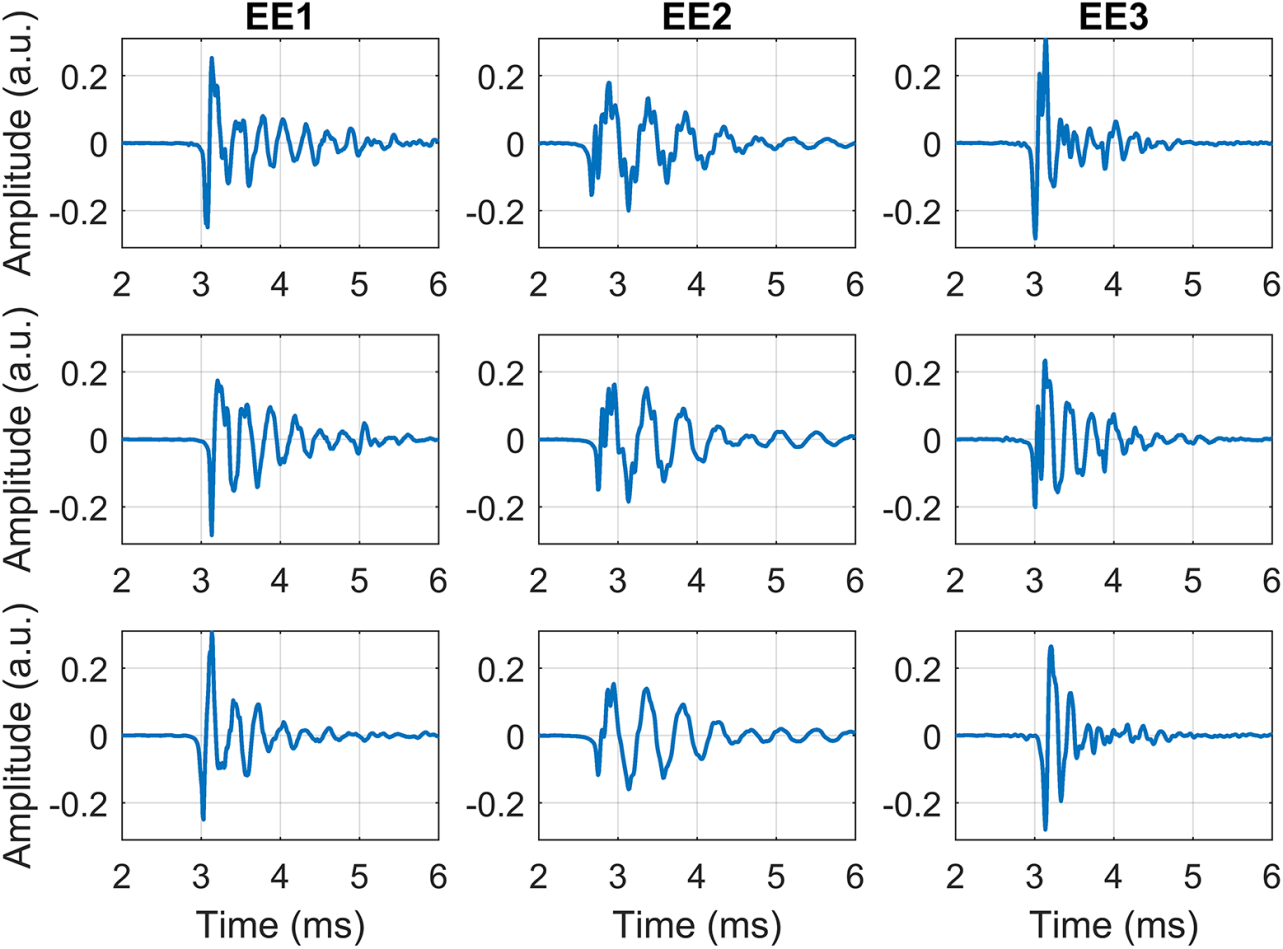
Illustrations of click waveforms. Illustrations of waveforms of three clicks for each of the three echolocators.

All analysis were done using Matlab (The Mathworks, Natick, USA) and custom written routines. The frequency content of the click, the spatial form of the click (how the click power distributes in space), and the time-domain envelope of the click were considered. Individual clicks were extracted from audio files by peak detection, and isolating 300 samples prior to the peak and 399 post the peak. Visual inspection confirmed accurate selection of clicks as well as rejection of bad samples (e.g. clipping). The numbers of clicks that passed criteria for EE1 were 1280 (azimuth, 100cm), 1199 (azimuth, 40cm) and 885 (elevation), for EE2 they were 1577 (azimuth, 100cm), 1441 (azimuth, 40cm) and 1065 (elevation), and for EE3 they were 816 (azimuth, 100cm), 756 (azimuth, 40cm) and 560 (elevation). The average numbers of clicks for any spatial position for EE1, EE2 and EE3 were 40.5 (SD: 8.9), 49.2 (SD: 13.5) and 25.7 (SD: 5.2), respectively. Supporting S1 Table provides a complete breakdown. Average inter-click intervals for EE1, EE2 and EE3 were 526ms (SD: 112, median: 496), 738ms (SD: 58, median: 721) and 682ms (SD: 71, median: 672), respectively. Fig 1 illustrates waveforms of three representative clicks for each of the three echolocators.

It is important to note that the waveforms of clicks produced by a single echolocator are replicable, but that there is also some click to click variability. Correlation coefficients calculated in the time-domain between any two extracted clicks for EE1 were 0.98 (max), 0.14 (min), 0.77 (median), 0.74 (mean), for EE2 0.99 (max), 0.11 (min), 0.78 (median), 0.75 (mean), for EE3 0.96 (max), 0.12 (min), 0.53 (median), 0.54 (mean).

### Spectral content

Analyses on spectral content were carried out on recordings from the reference microphone, for all clicks for 100cm azimuth conditions for each echolocator. The reference microphone was always placed at 50cm straight ahead from the echolocator, even if the target microphone moved to various positions. For each click the discrete Fourier transform and spectrogram were calculated and used to obtain average power spectral density (PSD) estimates and spectrograms. Spectrograms were calculated using a Kaiser-Bessel window (*β* = 3) of 192 samples (2ms), and 191 samples overlap.

### Spatial distribution

The directivity pattern in the horizontal plane (ϕ = 0°, θ = {−90°,−80°,…,180°}) and in the vertical plane (ϕ = {−40°,−30°,…,−140°}, θ = 0°) was evaluated. To suppress unsystematic click-to-click variation, the power of signals measured at the target microphone were normalized by the corresponding signal powers measured at the reference microphone. As several clicks were produced at each angular position the mean power ratio was calculated for each position as shown in Eq 1.

$$D(\theta ,\phi )=\frac{1}{N(\theta ,\phi )}\;\sum_{n=1}^{N(\theta ,\phi )}\frac{\sum_{t=1}^{T-1}{C(t)}_{n,sig}^{2}}{\sum_{t=1}^{T-1}{C(t)}_{n,ref}^{2}}$$ (1)

In Eq 1, which calculates the total power directivity pattern as the mean ratio of target to reference powers at each angular position, *C*(*t*)_*n*,*sig*_ is the n^th^ click recorded at the target microphone and *C*(*t*)_*n*,*ref*_ is the same click recorded at the reference microphone. *N*(*θ*,*ϕ*) is the total number of clicks at a given azimuth and elevation position, and *T* is the click duration in samples. Subsequently, azimuthal directivity patterns were fitted in order to mathematically describe them. A sufficient fit was found to be a modified cardioid fit, i.e. pure cardioid (numerator) modified by an ellipse (denominator). This is given in Eq 2, where α and β are constants which varied between echolocators, and that were estimated by performing a non-linear least squares fit with a trust-region algorithm implemented in the Matlab optimization toolbox [18].

$$R(\theta )=\frac{-(1+\cos\left(\theta \right))}{\sqrt{\alpha^{2}\cos^{2}\left(\theta \right)+\beta^{2}\sin^{2}\left(\theta \right)}}$$ (2)

A similar analysis was performed to investigate the directionality of different frequency components for more detailed reproduction of the clicks. Processing for this was similar to that used to form the total directivity patterns, but substituted the total click power for the power contained within specific frequency bands. This power can be estimated by summing the PSD estimate calculated over an appropriate range of frequencies as shown in Eq 3.

$$D(\theta ,\phi ,f_{hi},f_{lo})=\frac{1}{N(\theta ,\phi )}\;\sum_{n=1}^{N(\theta ,\phi )}\frac{\sum_{f=f_{lo}}^{f=f_{hi}}P{\left(f\right)}_{n,sig}}{\sum_{f=f_{lo}}^{f=f_{hi}}P{\left(f\right)}_{n,ref}}$$ (3)

In Eq 3, which calculates frequency-dependent directivity patterns as the mean ratio of target to reference power contained within a given frequency band at each angular position, *P*(*f*)_*n*,*sig*_ and *P*(*f*)_*n*,*ref*_ are the powers contained within each frequency f in the interval [*f*_*lo*_,*f*_*hi*_], for the n^th^ clicks recorded at the target and reference microphones, respectively.

### Time domain envelope

Typically the envelope of a signal is evaluated by low-pass filtering the signal, but this assumes a smoothly varying signal and performs poorly on the echolocators’ click by smoothing out their rapid-onset. To resolve this issue the click envelope was estimated by taking the absolute value of each click time sample, calculating peak positions, and interpolating the envelope between the peaks using a Piecewise Cubic Hermite Interpolating Polynomial (pchip) method implemented in Matlab [19]. Peaks were excluded if their height or prominence fell below 2% of the maximum peak height. This envelope estimate was then fitted with an exponential decay function mediated by a step function according to Eq 4.

E(t)=aexp⁡(−bt−c)H(t−c) (4)

In Eq 4, *H*(*t*) is the Heaviside step function, and *a*,*b*,*c* are rise magnitude (*a*), decay time constant (*b*), and onset time (*c*), i.e. constants which varied between echolocators, and that were estimated by performing a non-linear least absolute residual fit with a trust-region algorithm implemented in the Matlab optimization toolbox [18].

## Results

### Spectral content

**Fig 2:**
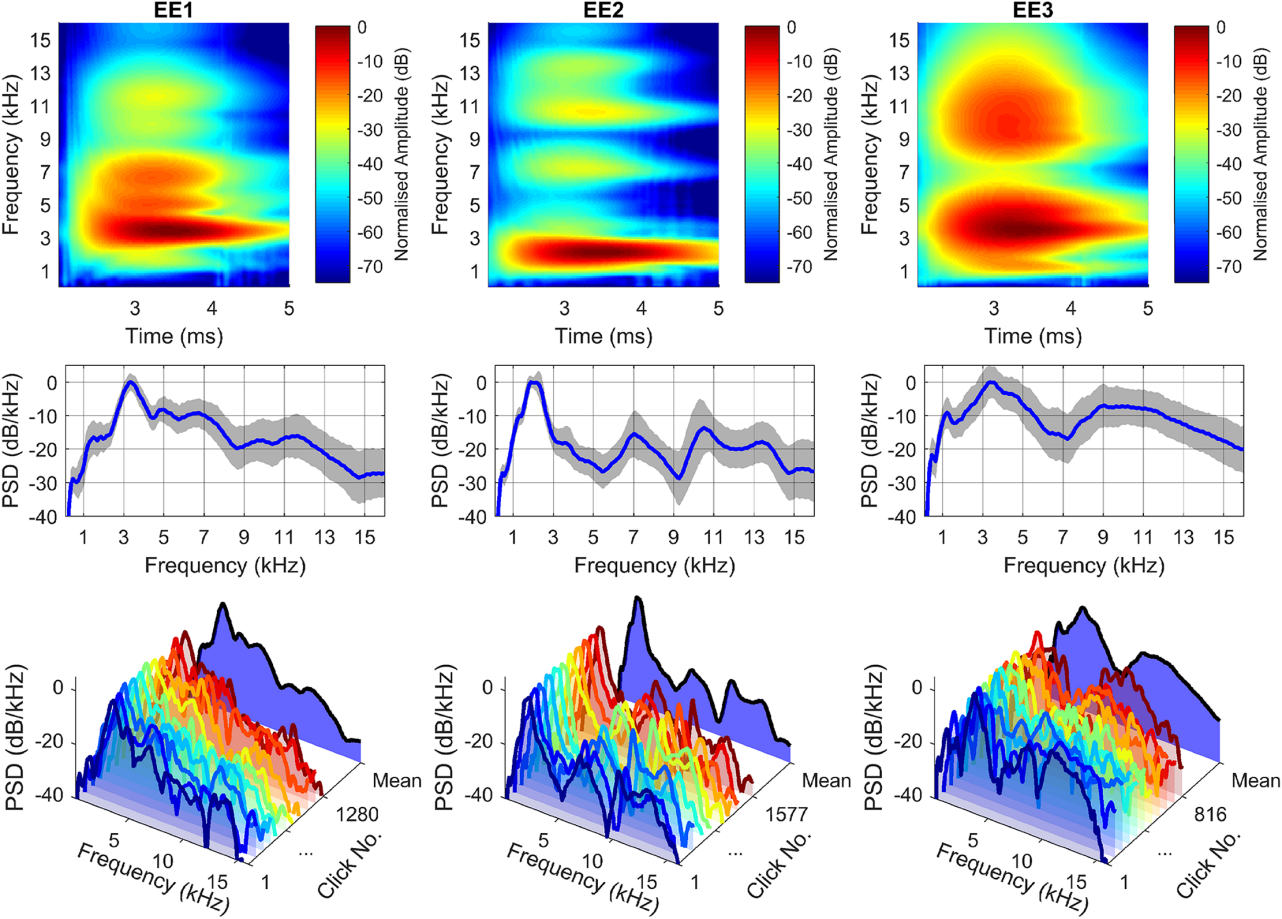
Frequency content of clicks for all echolocators. Top: Averaged spectrograms; Middle: Averaged power spectral density (PSD) plots; shaded regions denote 1 SD around the average; Bottom: Waterfall plots showing a set of PSD estimates for a subset of clicks and the average PSD estimate (black line at back).

Average spectrograms and PSD estimates shown in Fig 2 for EE1, EE2 and EE3 demonstrate that main frequency components are present and remain unchanged in frequency over the duration of the click. Grey shaded areas denote +/- 1SD around the average PSD (middle panels). To further illustrate the variation that each echolocator makes from click to click the foreground of the bottom plots of Fig 2 show a subset of the click PSD estimates for each echolocator, from which it can be seen that for EE1, while the main component at 3.39 kHz is present and remains relatively unchanged between clicks, there is variation in frequency content between the clicks elsewhere in the spectrum. In the background of the bottom plots of Fig 2 the averaged PSD estimates for the entire set of echolocator clicks are shown. Comparing PSD and spectrograms across individuals it is also visible that there are differences across EE1, EE2 and EE3 in terms of the spectral content of their clicks. Specifically, both EE1 and EE3 appear to have higher centre frequencies and broader spectral content when compared to EE2. Yet, peak frequencies for EE1-EE3 are all within 2-4kHz range, and all echolocators also had energy at ~10kHz. Even though energy at 10kHz was low compared to energy at peak, it was a local increase, as opposed to a smooth drop-off from peak towards the high end of the spectrum, for example. Table 1 provides information about peak frequencies from Fig 2 in numerical format. It is interesting to note, that within our three participants those who have emissions with higher frequency content had obtained better angular resolution in previous behavioural tests. For example, angular resolution thresholds for EE1 vs. EE2 based on [17] were 4° and 9° respectively, and for EE3, EE1 and EE2 based on [5] were 1.2°, 1.4° and 7.6°, respectively.

**Table 1:** Variability of spectrum peaks in frequency and amplitude.

	Dist. (cm)	Peak	Peak Frequency (kHz)			Peak Amplitude (dB/kHz)		
EE1	40	1	3.39	±	0.16	-17.2	±	1.6
		2	5.05	±	0.30	-25.4	±	2.4
		3	6.68	±	0.54	-25.7	±	2.5
		4	9.09	±	0.74	-34.4	±	4.3
		5	11.49	±	0.52	-33.6	±	4.1
	100	1	3.37	±	0.16	-17.1	±	1.5
		2	4.99	±	0.27	-25.3	±	2.4
		3	6.67	±	0.56	-25.8	±	2.4
		4	9.09	±	0.68	-34.7	±	3.9
		5	11.49	±	0.53	-34.2	±	3.9
EE2	40	1	2.07	±	0.22	-14.9	±	1.0
		2	7.17	±	0.52	-32.6	±	4.6
		3	10.69	±	0.50	-30.1	±	5.1
		4	13.35	±	0.54	-34.7	±	4.8
	100	1	1.95	±	0.18	-14.7	±	0.8
		2	7.16	±	0.57	-32.8	±	4.4
		3	10.73	±	0.58	-32.8	±	5.4
		4	13.51	±	0.66	-36.1	±	4.5
EE3	40	1	3.63	±	0.56	-19.2	±	2.4
		2	9.94	±	1.04	-25.2	±	3.2
	100	1	3.87	±	0.63	-19.9	±	2.3
		2	9.99	±	1.12	-25.7	±	2.9

### Spatial distribution

**Fig 3:**
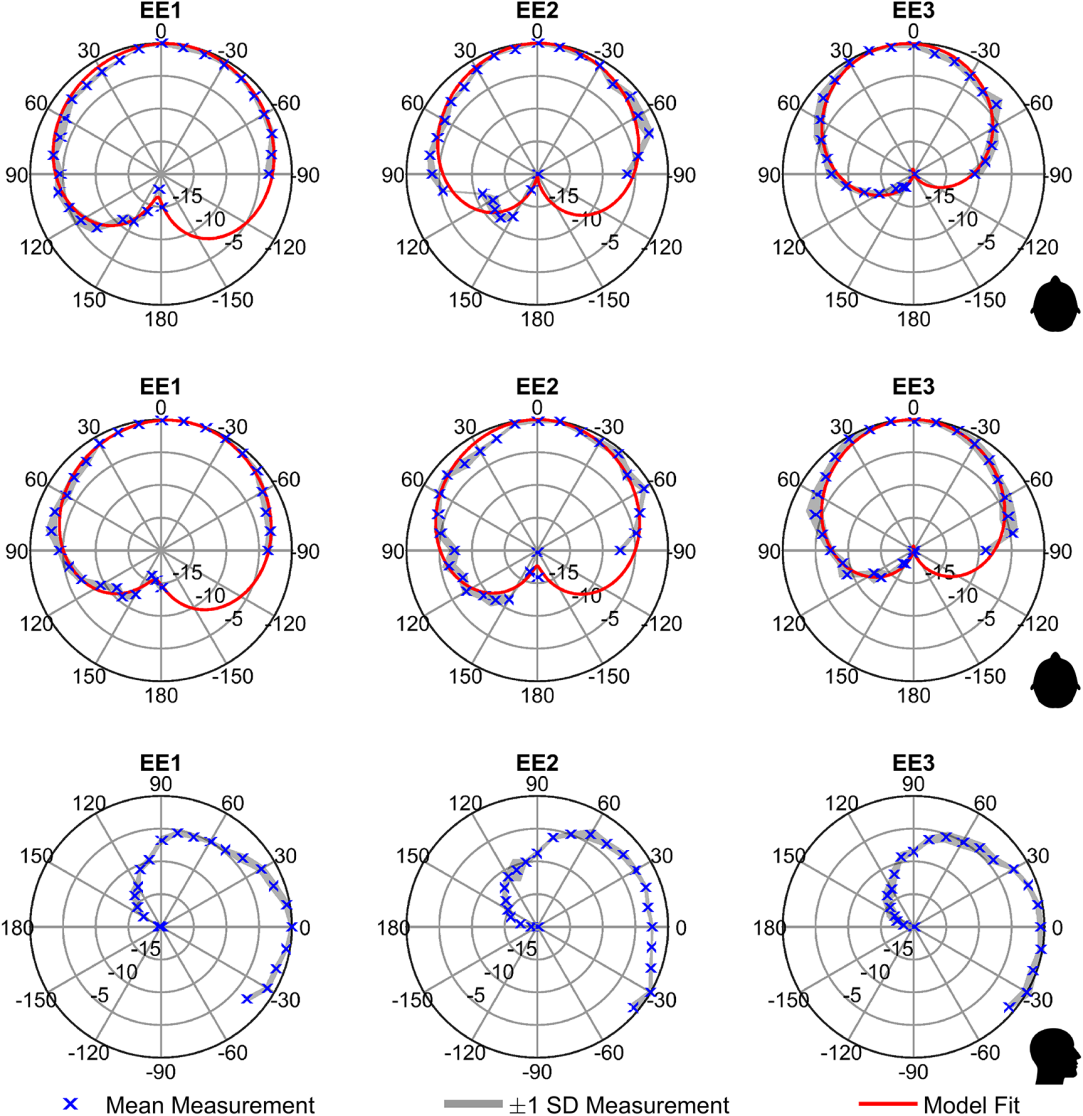
Directivity diagrams. Top row: Azimuth directivity diagrams for EE mouth-clicks at 40cm. Markers indicate average of measured data; shaded regions denote 1 SD around the average; red line is fit of a modified cardioid. Middle row: Azimuth directivity diagrams for EE mouth-clicks at 100cm. Symbol and colour coding as in top row. Bottom row: Elevation directivity diagrams for EE mouth-clicks. Markers indicate average of measured data; shaded regions denote 1 SD around the average.

Fig 3 top and middle rows present the average directivity diagrams produced for the echolocators in the horizontal plane for overall sound energy at 100cm and 40cm respectively using Eq 1. These figures are relative intensity plots, normalised to the maximum average intensity found in each data set. The figures show that click intensity is at a maximum in the forward direction (*θ* = 0°) and stays fairly constant within a 60° cone emanating from the mouth, and smoothly and gradually decreases towards the reverse direction (*θ* = 180°). Patterns are left-right symmetric. These patterns were fitted with the modified cardioid given in Eq 2. Fig 3 bottom row presents the diagrams produced for the echolocators in the vertical plane for overall sound energy at 40cm. The vertical plane directivity diagrams show that the behaviour in the vertical plane is similar to that in the horizontal plane, but with more variation (likely due to the shape of the head which is not front-back symmetric). Data are available in supporting S2 Table.

**Fig 4:**
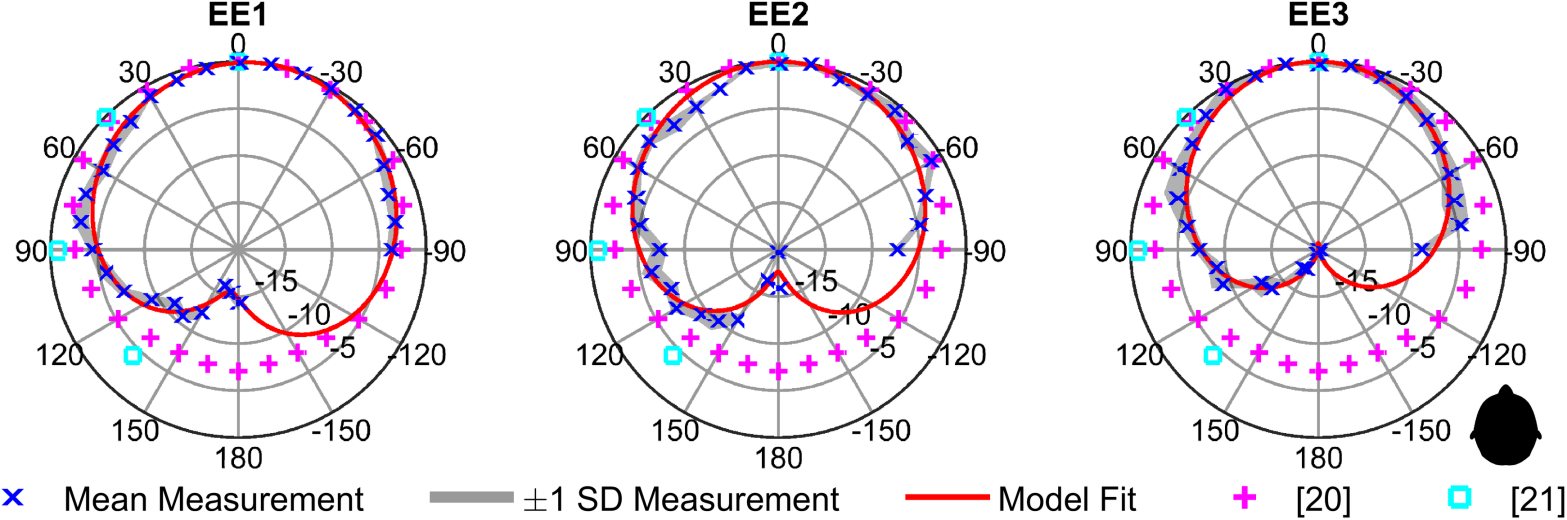
Directivity diagrams. Azimuth dependent directivity diagrams for speech based on [20] (magenta) and [21] (cyan) and for clicks from EE1-EE3 (red lines and blue symbols; plotted as in Fig 3). All measurements from 100cm.

For comparison, Fig 4 shows directivity patterns for speech based on data published in [20], and [21], and superimposed the directivity patterns of clicks. It is evident that directivity of clicks exceeds directivity of speech.

**Fig 5:**
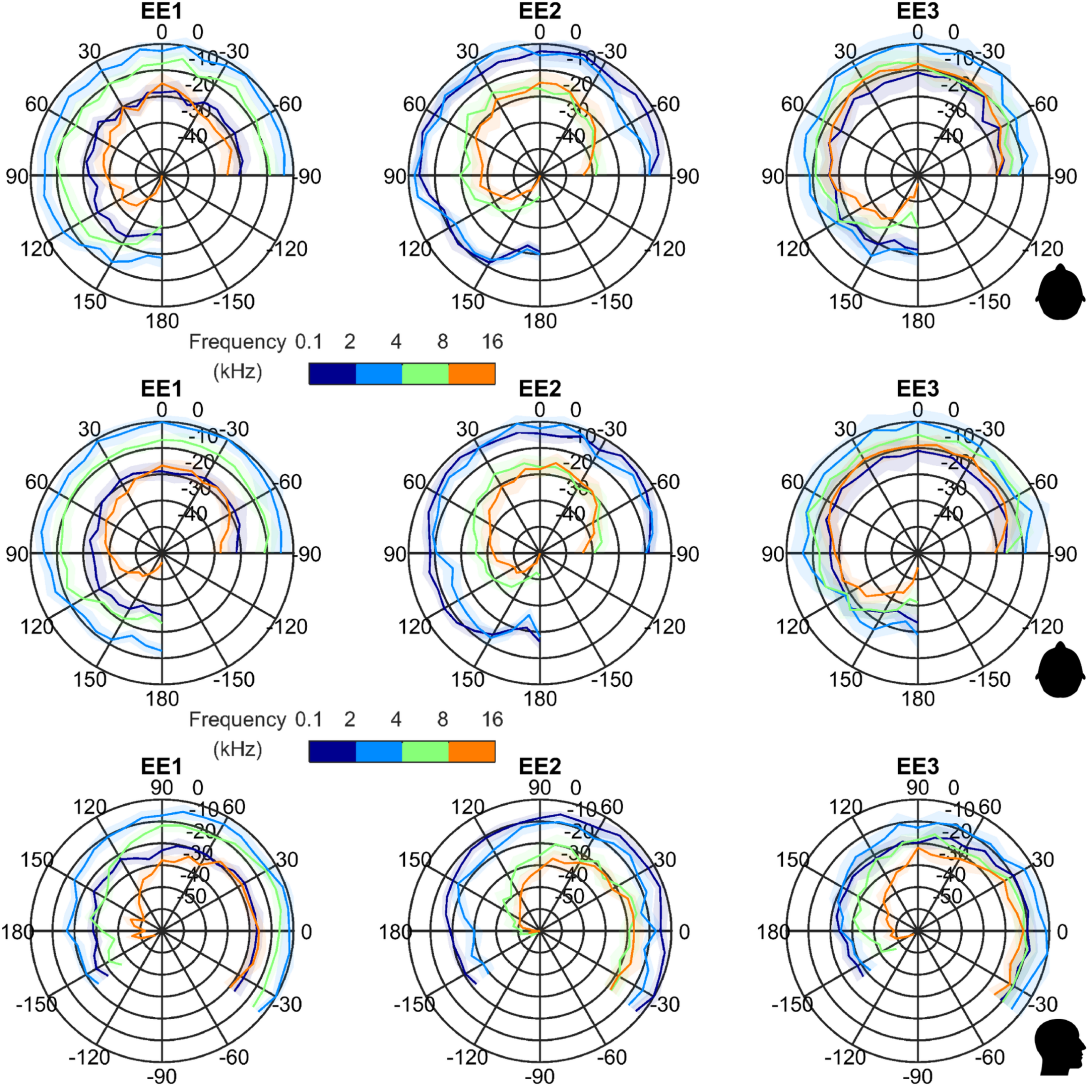
Frequency dependent directivity diagrams. Top row: Azimuth frequency-dependent directivity diagrams for EE mouth-clicks at 40cm. Lines indicate average of measured data; shaded regions denote 1 SD around the average; different colours denote different frequency bands. Middle row: Azimuth frequency-dependent directivity diagrams for EE mouth-clicks at 100cm. Line and colour coding as in top row. Bottom row: Elevation frequency-dependent directivity diagrams for EE mouth-clicks. Line and colour coding as in top row.

Fig 5 top, middle, and bottom rows show frequency-dependent directivity patterns for horizontal and vertical planes respectively (horizontal measured at 40cm, top, and 100cm, middle). One can see that EE1 exhibits higher click directivity in azimuth for the high frequency band compared to the low frequency band. These figures also show that EE3 exhibits higher click directivity in elevation for the high frequency band compared to the low frequency band. Data are available in supporting S3 Table.

### Envelope

**Fig 6:**
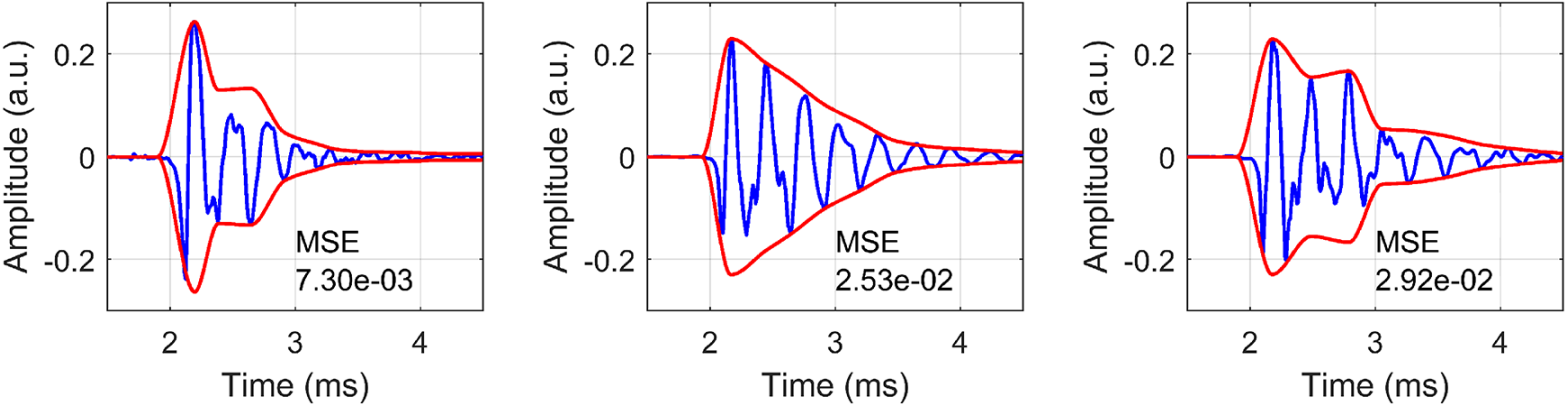
Time domain envelope. Figures show envelope estimate for three EE1 Clicks, along with the mean squared error (MSE) of the estimate.

Fig 6 shows three sample EE1 clicks along with the estimated envelope, demonstrating that the implemented algorithm performs well in estimating the envelope. The median mean squared error (MSE) of the envelope estimates for each echolocator were .0133 (EE1), .0084 (EE2) and .0485 (EE3).

Subsequently, the envelope function in Eq 4 was fitted to envelope estimates. R^2^ values of fits for EE1 were .9989 (median), .9996, (max), .9887 (min), .9987 (mean), for EE2 they were .9983 (median), .9995 (max), .9885 (min), .9979 (mean), for EE3 they were .9969 (median), .9992 (max), .5757 (min), .9958 (mean). Table 2 shows median estimates for rise magnitude (*a*), decay time constant (*b*), and onset time (*c*) for EE1-EE3 based on envelope fits. Based on these results duration of EE1, EE2 and EE3’s clicks is 2, 3 and 2ms, respectively (i.e. time for sound energy to drop to 5% of its original magnitude), or 3, 4 and 3 ms (time to drop to 1% of original magnitude).

**Table 2:** Synthetic click parameters for EE1, EE2, and EE3.

	f [kHz]	N	*ϕ*	a	b	c [ms]	α	β
**EE1**	3.54 , 5.30 , 6.93 , 9.97 , 11.88	6.58 , 2.68 , 2.49 , 0.868 , 1.00	1.59 , 1.60 , 1.65 , 1.72 , 1.39	0.388	1.57x10^3^	1.10	0.130	0.282
**EE2**	2.20 , 7.20 , 10.78 , 13.26	8.40 , 1.22 , 1.68 , 0.98	1.46 , 1.57 , 1.57 , 1.53	2.23	1.05x10^3^	1.97	0.101	0.185
**EE3**	3.67, 10.01	5.21, 2.70	1.59, 1.56	6.57	1.56x10^3^	2.03	0.963	0.104

### Click synthesis

Results gained from click analysis were used to derive artificial clicks. The aim was not to approximate a single click, but rather to create a click that is typical of the general set for EE1, EE2, and EE3 at various azimuth angles. The synthetic click for EE3 is less representative than the synthetic click for EE1 and EE2 due to the larger variation of EE3’s main frequency components.

The clicks were modelled as sum of monotones mediated by an envelope function *E*(*t*) in a process developed from [22]. Specifically, Eq 5 was used to build synthetic clicks by extracting typical click parameters from the database of clicks. The parameters that were extracted for each echolocator were coefficients of the envelope function *E*(*t*) (rise magnitude (*a*), decay time constant (*b*), onset time (*c*)), monotone centre frequencies (*f*), monotone magnitudes (*N*), monotone phases (*ϕ*), and modified cardioid parameters (α and β). All parameter values are given in Table 2. Eq 5 provides the monotones model for a synthetic click.

$$C_{synth}(t)=-R(\theta )\;E(t)\sum_{i=1}^{5}N_{i}\;\cos\left(2\pi f_{i}t+\phi_{i}\right)$$ (5)

To extract monotone centre frequencies and magnitude parameters from the click database, peak frequencies and amplitudes were extracted for each click from the PSD estimate within a set of manually-selected frequency bands (EE1: 2–4.5kHz, 4.5–5.8 kHz, 5.8–8.2kHz, 8.2–11 kHz, 11-13kHz; EE2: 1-3kHz, 5.5-9kHz, 9–12.4kHz, 12.4-16kHz; EE3: 2-6kHz, 7.5-12kHz). The median value of frequency and amplitude for each band were then used. The envelope function parameters were determined by fitting the function to envelope estimates, and then using median values of the parameter distribution obtained from these fits. Cardioid parameters α and β were estimated for each echolocator by performing a non-linear least squares fit with a trust-region algorithm implemented in the Matlab optimization toolbox [18] (compare section 2.4. Description/Analysis of Clicks).

**Fig 7:**
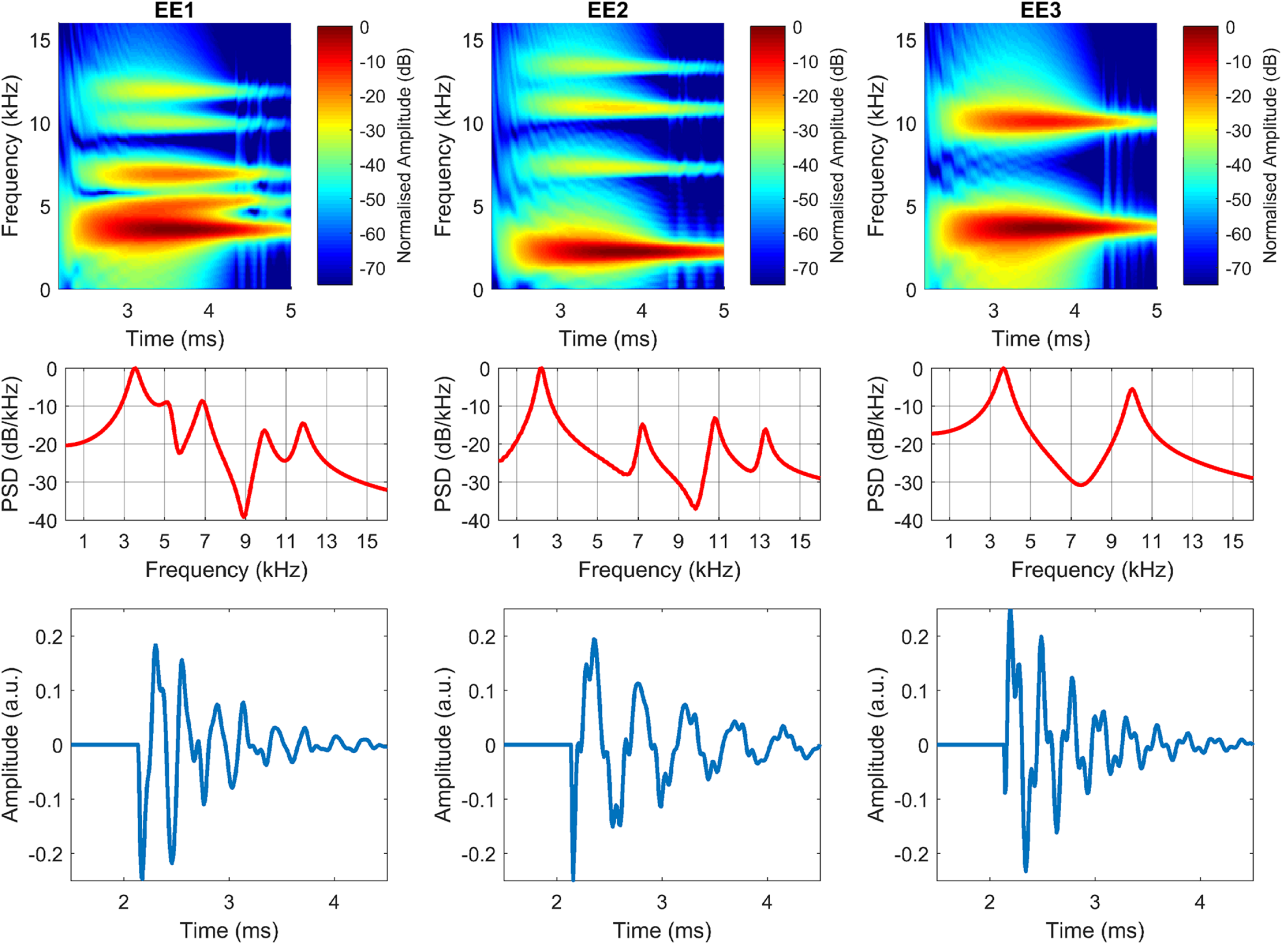
Click synthesis. Synthetic clicks plotted for EE1 (left), EE2 (middle), and EE3 (right) in the frequency-time-domain (top), frequency-domain (middle), and time-domain (bottom).

Fig 7 shows synthetic clicks for EE1, EE2, and EE3 at 0° azimuth. Matlab code to synthesize the clicks is available in supporting S1 Code.

## Discussion

The current report provides the first description of the spatial characteristics (i.e. beam pattern) of human echolocation transmissions based on measurements in three blind human echolocators, as well as spectro-temporal descriptions at a level of detail not available before. A model to generate the transmission as a function of angle for each echolocator is also provided. We found that acoustics of transmissions were consistent across echolocators in particular with respect to duration (~3ms) and directionality. We also found that directionality of clicks exceeded directionality of speech (as reported by [20] and [21]). Peak frequencies varied across echolocators, but nonetheless were all within the 2-4kHz range, and all echolocators also had energy at ~10kHz. Even though energy at 10kHz was low compared to energy at peak, it was a local increase, as opposed to a smooth drop-off from peak towards the high end of the spectrum, for example. EE1, EE2 and EE3 produced clicks with average inter-click intervals of 526ms, 738ms and 682ms, respectively. The analysis and synthesis methods we have used here are new (i.e. sum of monotones modulated by a decaying exponential with angular attenuation provided by a modified cardioid), and only possible because of the detailed measurements we had obtained. The models fit emissions well and are a viable method for synthetic generation.

Interestingly, within our three participants those who had emissions with higher frequency content had obtained better angular resolution in previous behavioural tests. Angular resolution thresholds for EE1 vs. EE2 based on [17] were 4° and 9° respectively, and for EE3, EE1 and EE2 based on [5] were 1.2°, 1.4° and 7.6°, respectively. This is in agreement with previous studies that have found relationships between spectral features of clicks and performance, e.g. [7].

The fact that echolocators in our study consistently made clicks ~3ms duration does not imply that this would be an ‘optimal’ duration for human echolocation. Rather, 3ms might be the minimum duration humans can achieve considering their vocal apparatus and the tissues involved in generating the click. We may speculate that perhaps, in general, briefer emissions may present an advantage for expert human echolocators, for example in terms of reproducibility, immunity to noise, and/or in terms of spatial resolution.

Echolocators in our study had been instructed to make clicks as they usually would during their everyday activities. The room was empty. In this way the task was a ‘non-target’ task, i.e. echolocators did not actively echolocate a target. Bats can adjust their emissions dynamically, for example, some species may shift spectro-temporal aspects of their calls (i.e. duration, spectrum, pulse rate) pending on the environmental conditions [10–14], or they may adjust the direction and/or width of their sound beam when they lock onto a target [9,10,15,16]. The question arises if blind human expert echolocators may adjust their clicks as well. Our current report does not speak to this issue because we only measured clicks in a ‘non-target’ setting. Nonetheless, in regards to the beam pattern it is important to point out that the anatomy of the human head, mouth and lips poses severe limitations on the flexibility of the width of the spatial distribution of a click (and speech as well). On the other hand, the direction into which a click is pointed can be varied easily by head-rotation. In regards to spectro-temporal characteristics there is some flexibility, for example by changing the shape of the lips or simply clicking at a higher rate (i.e. reducing inter click intervals). Therefore, based on research in bats and our finding that the click beam pattern is oriented forwards with energy fairly constant within a 60° cone, we might for example expect that people exhibit more variability in head rotation angle when they scan for a target as compared to when they approach a target, and changes in head rotation behaviour might be accompanied by changes in click peak frequency or clicking rate. In sum, our results suggest that future research should address dynamic emission adjustments in people.

There have been previous approximations of duration and peak frequencies of human echolocation emissions in the straight ahead direction [6,7,8]. These investigations did not provide any directivity or rate measurements and range of estimates was wide (duration: 3-15ms; peak frequencies: 2-8kHz), likely due to the fact that samples included sighted people who do not use echolocation on a daily basis. Rojas and colleagues [8] also commented on signal properties such as replicability, and immunity to noise, but they did not provide empirical data to support arguments made. Our analysis of inter-click correlations suggests that indeed the clicks made by human expert echolocators have a high degree of replicability. Importantly, in bats it has been shown that spatio-temporal properties of the emission can explain aspects of echolocation behaviour, e.g. [9,10] and even properties of neural activity, e.g. [23]. The same might be possible in people, highlighting the importance of the data reported here for investigating human echolocation in a hypothesis driven way.

Human biosonar consists not only of the transmission (e.g. mouth click), but also of the reception of the resultant sound through the ear. It follows, therefore, that only combining these two elements will permit precise predictions for echolocation performance, for example, based on signal strength. One might expect that target detection should be better at angles with stronger received signal strength as compared to angles with lower received signal strength. The model of the human biosonar emission we provide here, together with existing HRTF databases, makes future hypothesis-driven work of this kind possible. There have been prior studies trying to measure precision and acuity of human echolocation, but these have exclusively focused on performance in the median plane (see [2–4] for reviews). The current results clearly suggest that there is merit in characterizing performance at farther angles also.

The data presented here are a basis to develop synthetic models of human echolocation, which will help understanding the link between physical principles and human behaviour. Understanding characteristics of click echoes from various objects could be used to understand human echolocation behaviour in tasks such as localising or recognising an object, navigating around it etc. To undertake this type of work large amounts of data are required (for example, a radar reflectivity measurement of a single object typically requires thousands of measurements). These are impractical to ask from human subjects. One could also build instrumentation (e.g. loudspeakers) that can create beam patterns either matching those of human echolocators, or not, which can then be used to systematically measure effects of beam patterns on performance. Building of synthetic models and instrumentation requires understanding of the properties of the click waveform itself and its spatial distribution after transmission, which is the purpose of this paper.

Echolocation can provide humans with information about the distal environment that is not limited to spatially localising an object. Specifically, the same echolocation process is used to reveal information about size, shape and material of objects as well as their spatial location (for reviews see [2,3,4]). Developers of artificial sonar and/or radar systems might therefore benefit from our results via use of synthetic models because they might be useful for development of artificial systems that provide multifaceted information about the distal environment.

Human sonar emissions are well within the audible spectrum. In contrast, echolocating bats or toothed whales can produce emissions in the ultrasonic range (>20kHz). Whilst frequency sweeps are a common emission in bats, some bat species also use clicks and demonstrate remarkable echolocation abilities [24]. Based on physics, higher sound frequency translates into better spatial resolution. As such, one might suspect human echolocators to be at a disadvantage compared to bats based on acoustics of the emissions alone. Nonetheless, people have shown to be able to resolve lateral position of objects separated by less than 2°, with best performers having shown thresholds between 1.2° and 1.9° [5]. This compares favourably to the acuity of some bats when measured in a similar way [25]. Again, the emission models we provide here in combination with existing HRTF data bases can be used to build echo-acoustic models to investigate how this human level of performance might be possible.

Virtual echo-acoustic models permit stimulus control not possible in natural environments and can therefore be a useful tool for understanding echolocation processes, e.g. [26,27]. For humans in particular they are also ideal to investigate neural processes in environments that are not suitable for ‘real’ echolocation due to constraints on space and/or body movement (e.g. fMRI, MEG, EEG) [28]. Yet, at present, virtual echo-acoustic models for investigating human echolocation have no empirical basis for their choice of directional propagation of click emissions. It follows that models of emissions such as those provided here are required to use accurate virtual echo-acoustic models to further advance understanding of human echo-acoustic processing.

## Supporting information

S1 Table Numbers of clicks broken down by participant (EE1, EE2, EE3), condition (azimuth 40cm, azimuth 100cm, elevation) and angle. Labelling of angles as in Fig 3, Fig 4 and Fig 5.
(XLSX)

S2 Table Elevation directivity data for EE mouth-clicks broken down by participant (EE1, EE2, EE3) and angle. Labelling of angles as in Fig 3 and Fig 5.
(XLSX)

S3 Table Frequency-dependent directivity data broken down by participant (EE1, EE2, EE3), condition (azimuth 40cm, azimuth 100cm, elevation) and angle. Labelling of angles as in Fig 3, Fig 4 and Fig 5.
(XLSX)

S1 Code Matlab code to synthesize clicks for each individual (EE1, EE2, EE3). (ZIP)
